# Alternating Current Electrospinning of Polycaprolactone/Chitosan Nanofibers for Wound Healing Applications

**DOI:** 10.3390/polym16101333

**Published:** 2024-05-09

**Authors:** Jon Andrade del Olmo, Petr Mikeš, Nikifor Asatiani, José María Alonso, Virginia Sáez Martínez, Raúl Pérez González

**Affiliations:** 1 i+Med S. Coop., Alava Technology Park, Albert Einstein 15, nave 15, 01510 Vitoria-Gasteiz, Spain; jalonso@imasmed.com (J.M.A.); vsaez@imasmed.com (V.S.M.); rperez@imasmed.com (R.P.G.); 2 Department of Physics, Faculty of Science, Humanities and Education, Technical University of Liberec, Studentská 1402/2, 461 17 Liberec, Czech Republic

**Keywords:** electrospinning, polycaprolactone, chitosan, nanofibers, wound healing, tissue regeneration

## Abstract

Traditional wound dressings have not been able to satisfy the needs of the regenerative medicine biomedical area. With the aim of improving tissue regeneration, nanofiber-based wound dressings fabricated by electrospinning (ES) processes have emerged as a powerful approach. Nowadays, nanofiber-based bioactive dressings are mainly developed with a combination of natural and synthetic polymers, such as polycaprolactone (PCL) and chitosan (CHI). Accordingly, herein, PCL/CHI nanofibers have been developed with varying PCL:CHI weight ratios (9:1, 8:2 and 7:3) or CHI viscosities (20, 100 and 600 mPa·s) using a novel alternating current ES (ACES) process. Such nanofibers were thoroughly characterized by determining physicochemical and nanomechanical properties, along with wettability, absorption capacity and hydrolytic plus enzymatic stability. Furthermore, PCL/CHI nanofiber biological safety was validated in terms of cytocompatibility and hemocompatibility (hemolysis < 2%), in addition to a notable antibacterial performance (bacterial reductions of 99.90% for *S. aureus* and 99.91% for *P. aeruginosa*). Lastly, the enhanced wound healing activity of PCL/CHI nanofibers was confirmed thanks to their ability to remarkably promote cell proliferation, which make them ideal candidates for long-term applications such as wound dressings.

## 1. Introduction

Electrospinning (ES) is a well-known technique used to fabricate tunable polymeric fibers ranging from several nanometers to micrometers by applying high-voltage electric fields [1]. Electrospun fibers possess high flexibility, versatility, porosity and surface/volume ratio with a controllable structure that makes them suitable for application in the wound healing biomedical field [2,3]. These advanced fibrous dressings are suitable for the treatment of acute and chronic wounds due to their capability of imitating native extracellular matrix (ECM) and promoting biological processes (e.g., cell migration, adhesion, proliferation and differentiation) [4,5].

Traditional dressings (gauzes or bandages) possess limited bioactive properties, the inability to eliminate exudate or maintain moisture, and the difficulty of removal after application [6,7]. Fortunately, nanofiber-based bioactive dressings do not need secondary dressings and possess enhanced biocompatibility, biodegradability, adhesion, permeability, exudate absorption, moisture retention and easy changing without damage [7]. Even more, bioactive wound dressings could provide antibacterial and anti-inflammatory properties for improved tissue regeneration [8].

Nowadays, nanofiber-based bioactive wound dressings are mainly fabricated with a combination of natural and synthetic polymers [9]. While natural polymers provide the benefits of easy biodegradation and excellent biocompatibility, synthetic polymers contribute controllability and reliable mechanical strength [10]. Among synthetic polymers, FDA-approved biocompatible, biodegradable and bioresorbable polycaprolactone (PCL) is one of the most widespread used co-spinner [11]. However, PCL lacks bioactive properties and possesses moderate wound exudate absorption and water retention capabilities.

In this regard, PCL-based dressings’ drawbacks can be improved by combining them with a wide range of natural biopolymers [12]. Among these, alginate, cellulose, hyaluronic acid and chitosan polysaccharides are known to effectively combine with PCL to produce multifunctional dressings [13]. Chitosan (CHI) is a partially deacetylated and positively charged derivative of chitin with random mixtures of β-(1–4) and *N*-acetyl D-glucosamine units, which has aroused biomedical experts’ attention due to its biocompatibility, biodegradability, hemostatic ability, angiogenesis, immunoregulation, and antibacterial and anti-inflammatory properties, among others [14,15].

In this sense, PCL/CHI nanofibers combine the powerful advantages of both polymers and avoid the complexity of ES pure CHI. In fact, the fabrication of pure CHI nanofibers is considered a challenge due to their polycationic nature and hydrophilicity, which result in insufficient chain entanglement and, consequently, a restricted spinning ability [16]. Such fabrication of PCL/CHI nanofibers by ES can be carried out with solvent systems based on diluted acid combinations such as acetic acid (AA), formic acid (FA) and acetone (Ac), among others [17,18].

Regarding ES efficiency, a wide range of techniques have been investigated over the last decades to increase the productivity of nanofiber fabrication [19]. Alternating current ES (ACES) has emerged as a novel alternative approach to increase the productivity of typical direct current ES (DCES) [20,21]. In fact, although most of the published articles about ES only discuss DCES, its productivity is quite low for industrial applications [22]. Otherwise, the replacement of DCES with dynamic high-voltage ACES with the possibility of varying frequency and waveform results in higher productivity, even with laboratory setup [23]. That is why, in the last few years, PCL-based nanofibers have been fabricated by ACES with quite promising productivity results [24,25,26].

Moreover, it is well known that the electro-spinnability of PCL/CHI nanofibers can be greatly influenced by several factors, such as CHI quantity (or PCL:CHI weight ratio) [27,28] and intrinsic viscosity (affected by the molecular weight) [29,30]. To date, although some researchers have studied how CHI features have influenced the spinnability of PCL/CHI nanofibers by DCES [31,32,33], contradictory productivity results make imperative the development of PCL/CHI nanofibers by novel ACES and solvent systems. Even more, to the best of our knowledge, none of the investigations published so far fabricated PCL/CHI nanofibers by an ACES and FA:AA:Ac solvent system mixture or studied the influence of CHI factors on spinnability efficiency.

Considering all of the above, PCL/CHI nanofibers were synthesized using an uncommon ACES and FA:AA:Ac solvent system to obtain long-term biocompatible wound dressings with tunable physicochemical, mechanical and bioactive properties. For that purpose, PCL/CHI nanofibers were optimized using 9:1, 8:2 and 7:3 PCL:CHI weight ratios and 20, 100 and 600 mPa·s CHI viscosities. Such PCL/CHI nanofibers were thoroughly characterized by SEM, FTIR-ATR, XPS, contact angle, AFM, absorption capacity, and hydrolytic and enzymatic degradation techniques. Moreover, PCL/CHI nanofiber biocompatibility was assessed in terms of cytotoxicity and hemocompatibility, in addition to their antibacterial performance. Finally, wound healing properties of PCL/CHI nanofibers were evaluated by assessing cell proliferation. All in all, PCL/CHI nanofiber dressings could possess a promising future in the wound healing biomedical area.

## 2. Materials and Methods

### 2.1. Materials and Chemicals

Polycaprolactone (PCL, average M_n_ ≈ 80,000, 440,744, Sigma-Aldrich, St. Louis, MO, USA) and mushroom-source chitosan (CHI, Chibio Biotech, Qingdao, China) with three different viscosity values in 1% acetic acid (20 mPa·s, deacetylation degree of 98.23%; 100 mPa·s, deacetylation degree of 98.26%; 600 mPa·s, deacetylation degree of 98.22%) were used to synthesize PCL/CHI nanofibers. Formic acid (FA, 98%, 19,920–11,000), acetic acid (AA, 99.8%, 19,990–11,000) and acetone (Ac, 99.5%, 10,050–11,000) were purchased from Penta Chemicals Unlimited (Czech Republic) and used to prepare a solvent system. Lysozyme (from chicken egg white, enzymatic activity ≥ 40,000 U/mg, Sigma-Aldrich, L6876, MO, USA) and lipase (from *Pseudomonas cepacia*, enzymatic activity ≥ 30 U/mg, Sigma-Aldrich, 62309, MO, USA) enzymes were used for degradation tests.

### 2.2. Synthesis of PCL/CHI Nanofibers

PCL/CHI nanofibers were prepared by AC needle-electrospinning (Table 1). Briefly, PCL 10 wt% was dissolved in an FA:AA:Ac solvent system (1:1:1 weight ratio) for 3 h to prepare a pure PCL solution. Regarding PCL/CHI solutions, PCL concentration was maintained at 10 wt%, CHI content was adjusted to 2 wt%, and PCL:CHI weight ratios were varied from 9:1 to 8:2 and 7:3, in addition to three different mushroom sources of CHI (20, 100 and 600 mPa·s). Subsequently, each solution was placed into a 5 mL syringe coupled to an 18 G needle. In addition, flow rate was adjusted to 2 mL/h, and an input signal was created by an Instek GW MFG 2120 MA signal generator (30 Hz) applying a rectangular shape using a TREK 10/40A-HS amplifier (10 kV). Fibers were stored on a rotational collector with a fixed rotation speed of 40 rpm and fixed needle–collector distance of 15 cm.

### 2.3. Physicochemical and Mechanical Characterization

#### 2.3.1. Scanning Electron Microscopy (SEM)

High-resolution images of PCL and PCL/CHI electrospun nanofibers were achieved with a SEM FEI Quanta 250. The microscope worked under low-vacuum conditions at 5 kV, 120 Pa, 25 °C and 0% humidity, and images were acquired at 4000× magnification using a Large Field GaseoUs Secondary Electron detector (LFD). Fiber diameters were measured by employing an ImageJ (1.54f version, National Institutes of Health, Bethesda, MD, USA) image analyzer via the measurement of 100 observed nanofibers selected over different SEM images. Fiber normality data were verified primarily by the Shapiro–Wilk test, and homoscedasticity was verified by the Levene test (0.05 significance level). Since the assumptions of normality and homoscedasticity were violated, non-parametric analysis was used to determine the statistical significance of differences between the nanofibrous layers. Kruskal–Wallis’s test was performed followed by a Dunn’s post hoc test for multiple comparisons. Bonferroni correction was used for the adjustment of *p*-value when comparing multiple materials to pure PCL control. Numerical data were expressed as the mean ± standard deviation (SD), and fiber diameters were also expressed as median and interquartile range (IQR = Q3–Q1).

#### 2.3.2. Fourier Transform Infrared Spectroscopy by Attenuated Total Reflectance (FTIR-ATR)

An FTIR spectrometer (PerkinElmer Frontier, Shelton, WA, USA) in the ATR configuration was employed to determine the qualitative chemical composition of electrospun PCL and PCL/CHI nanofibers. Infrared spectra were recorded using a diamond crystal in the 800–4000 cm^−1^ range at a 4 cm^−1^ resolution and 32 scans/spectrum.

#### 2.3.3. X-ray Photoelectron Spectroscopy (XPS)

The quantitative composition of PCL and PCL/CHI electrospun nanofibers was obtained using an XPS—SPECS SAGE HR-100 equipped with a non-monochromatic radiation source (2·10^−7^ hPa, X-ray gun at 300 W and 12,500 V), a Mg/Al K_α_ anode and a PHOIBOS analyzer (100 mm radius).

#### 2.3.4. Atomic Force Microscopy (AFM)

The nanomechanical properties of electrospun nanofibers were obtained using a high-resolution AFM MultiMode 8-HR microscope (Bruker) and PeakForce Quantitative Nanomechanical Mapping module. A Scanasyst-Air probe (f = 70 kHz; k = 0.4 N/m) was employed for imaging 2 × 2 µm^2^ areas (0.5 Hz scan rate, 1 kHz peak force frequency, 300 nm amplitude and 1 nN setpoint). Micrographs were treated using Nanoscope 2.0 software.

#### 2.3.5. Contact Angle Measurements

Contact angle measurements to measure the wettability of nanofibers (*n* = 10) were performed with Dataphysics OCA 15 EC Neurtek (Eibar, Spain) optical equipment by dropping Milli-Q water (2 μL/drop).

#### 2.3.6. Absorption Capacity

Absorption capacity of nanofibers was determined using the free expansion absorptive capacity method according to the UNE-EN ISO 13726-1:2002 standard [34]. The 0.5 cm^2^ samples were weighed before testing (*W*_0_) and immersed into 40 times the mass equivalent of each sample of Test Solution A (8.298 g sodium chloride and 0.368 g calcium chloride dihydrate dissolved in 1 L of deionized water) at 37 ± 1 °C. Test Solution A has an ionic composition comparable to human blood serum or wound exudate [35]. After 30 min, nanofibers were removed, excessive solution was dripped off and samples were weighed again (*W_t_*). Absorption capacity (%) is expressed as the mean of the mass of solution retained per gram of sample (Equation (1)).

(1) $$Absorption\;capacity\;\left(\%\right)=\frac{W_{t}-W_{0}}{W_{0}}\cdot 100$$

#### 2.3.7. Hydrolytic and Enzymatic Degradation

PCL and PCL/CHI electrospun nanofiber degradation was monitored with respect to the weight loss of initially weighed electrospun nanofibers (*W*_0_), immersing each sample (0.5 × 0.5 cm^2^) separately in 10 mL of PBS (pH 7.4, 37 °C, hydrolytic), lysozyme (0.5 mg·mL^−1^ in PBS) and lipase (0.5 mg·mL^−1^ in PBS) solutions. Enzymatic solutions were not changed during the course of the experiment. Then, electrospun nanofibers were removed from each solution and weighed (*W_t_*) at specific times. The remaining electrospun nanofiber mass (%) was calculated according to Equation (2).

(2) $$Remaining\;nanofibers\;mass\;\left(\%\right)=\frac{W_{t}}{W_{0}}\cdot 100$$

### 2.4. Biocompatibility Test

#### 2.4.1. In Vitro Cytotoxicity

Cytotoxicity was evaluated by the extraction method based on the ISO 10993-5 guideline [36]. Extractor liquid, cell line, and positive and negative controls were DMEM culture medium (supplemented with 10% FBS), L929 (ECACC), polyurethane film with 0.1% zinc diethyldithiocarbamate (ZDEC), polyurethane film with 0.25% zinc dibuthyldithiocarbamate (ZDBC) and high-density polyethylene (HDPE), respectively. Some 100%, 50%, 25% and 12.5% extracts were obtained by incubating the compounds in culture media for 24 h at 37 °C. Some 1·10^5^ L929 cell suspensions were seeded in 96-well plates and incubated at 37 °C and 5% CO_2_ for 24 h. Later, extracts were added and incubated at 37 °C and 5% CO_2_ for 24 h. Finally, 50 µL of 3-(4,5-dimethylthiazol-2-yl)-2,5-diphenyltetrazolium bromide (MTT) solution was added and incubated again for 2 h. Cell viability (%) calculated by MTT assay was obtained using a spectrophotometer at 570 nm (Equation (3)).

(3) $$Cell\;viability\;\left(\%\right)=\frac{OD_{570\;tested\;nanofibers}}{OD_{570\;negative\;control}}\cdot 100$$

#### 2.4.2. In Vitro Hemocompatibility

Hemocompatibility of the nanofibers was assessed according to ISO 10993-4 standard [37]. Hemolysis was quantitatively evaluated measuring the release of hemoglobin from red blood cells either by destruction or through a partially damaged but intact cell membrane. This test was performed using a Biomaterial Haemolytic Assay Kit (K003, Batch No. 231016, HaemoScan, Groningen, The Netherlands) with human erythrocyte suspensions. Erythrocyte suspension was prepared using buffer solutions and 400× *g* centrifugation. Unexposed human blood was employed as a negative control, and silicon elastomer (SE), medical steel (MS) and nitrile rubber (Buna-N) were positive controls. Briefly, samples were immersed into 0.5 mL of erythrocyte suspension and incubated for 24 h at 37 °C. Then, the tubes were centrifuged at >4000× *g* for 1 min. After that, 20 µL was added to a 96-well plate and mixed with 180 µL of buffer. Hemoglobin concentration and hemolysis rate (or % hemolysis) were measured by spectrophotometry at 380, 415 and 450 nm using the Harboe method [38].

### 2.5. In Vitro Antibacterial Activity

The in vitro antibacterial activity of nanofibers was tested against *Staphylococcus aureus* (ATCC 6538) and *Pseudomonas aeruginosa* (ATCC 15442). Some 1 mL of 1·10^5^–1·10^6^ CFU/mL suspensions were put in contact with 1 × 1 cm^2^ samples and incubated at 37 °C for 24 h. Viable *S. aureus* or *P. aeruginosa* as Colony Forming Units (CFUs), bacterial death as reduction %, and antimicrobial activity as *log10* reduction or *R* were calculated by Equations (4) and (5).We

(4) $$Bacterial\;reduction\;\left(\%\;death\right)=\frac{B-A}{B}\cdot 100$$

(5) $$log10\;reduction\;\left(R\right)=log10\;\left(B\right)-log10\;\left(A\right)$$

where *A* is the average of viable cells (CFU) after contact time with PCL/CHI nanofibers, and *B* is the number of average viable cells (CFU) in control samples after 24 h of contact time. The control corresponds to *S. aureus* or *P. aeruginosa* suspensions in the absence of tested product.

### 2.6. In Vitro Wound Healing Cell Proliferation Assay

Wound healing properties were evaluated in vitro by assessing cell proliferation activity induced by nanofibers. PCL and PCL/CHI nanofibers were deposited on cellulose cardboard for this test. Cellulose cardboard without nanofibers was used as a control. Human dermal fibroblasts (HDFs, Innoprot, P60108, Derio, Spain) isolated from human skin were used as an experimental system. HDF cells were cultured for 48 h (37 °C, 5% CO_2_ and 95% humidity) in a 12-well chamber containing nanofibers for confocal laser scanning microscopy (CLSM, Zeiss LSM700, Jena, Germany) applications. Then, cells were stained using an SYTO9 fluorescent marker to intercalate into cell DNA and, subsequently, green-stained cells were visualized by CLSM. Images were taken using 2.5× magnification, and florescence was quantified using ImageJ computer software (1.54f version).

### 2.7. Statistical Analysis

Quantitative variables of assays are represented as means ± standard deviations for a minimum of *n* = 3 replicates per group. Multiple comparisons between groups were performed using one-way ANOVA, followed by a post hoc Tukey’s test with a *p* < 0.05 significance level threshold.

## 3. Results and Discussion

### 3.1. Physicochemical Characterization

**Figure 1:**
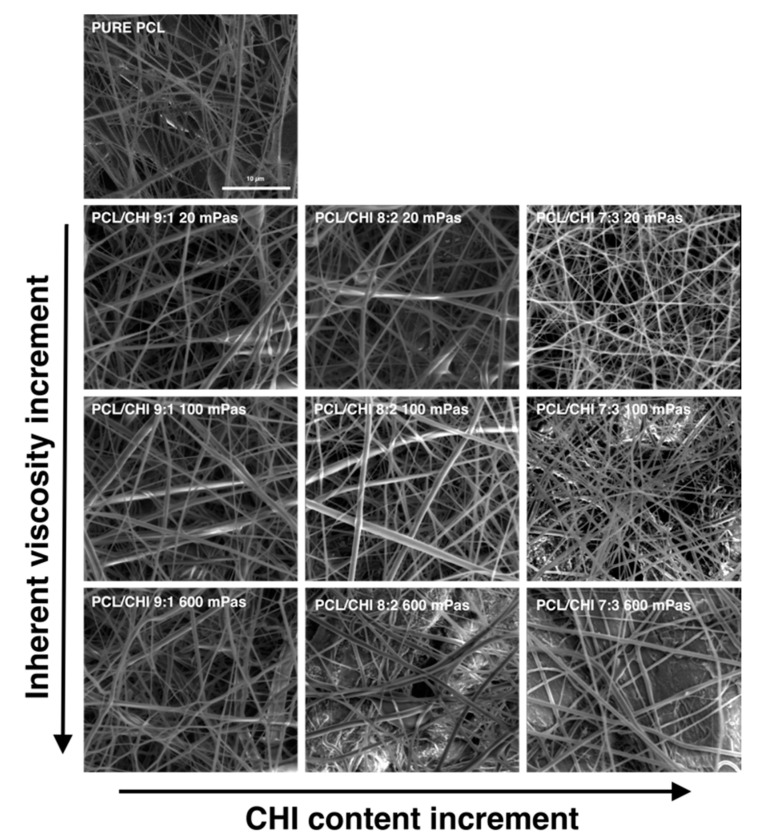
Representative SEM images of the fabricated PCL and PCL/CHI nanofibers obtained with different PCL-to-CHI ratios (9:1, 8:2 and 7:3) and CHI inherent viscosities (20, 100 and 600 mPa·s).

First of all, the morphology and structure of fabricated PCL and PCL/CHI fibrous biomaterials were studied by scanning electron microscopy (SEM) images (Figure 1). SEM is capable of producing detailed images with a maximum resolution of 1 nm and is frequently useful to determine nanofiber diameter or alignment, among other aspects.

**Figure 2:**
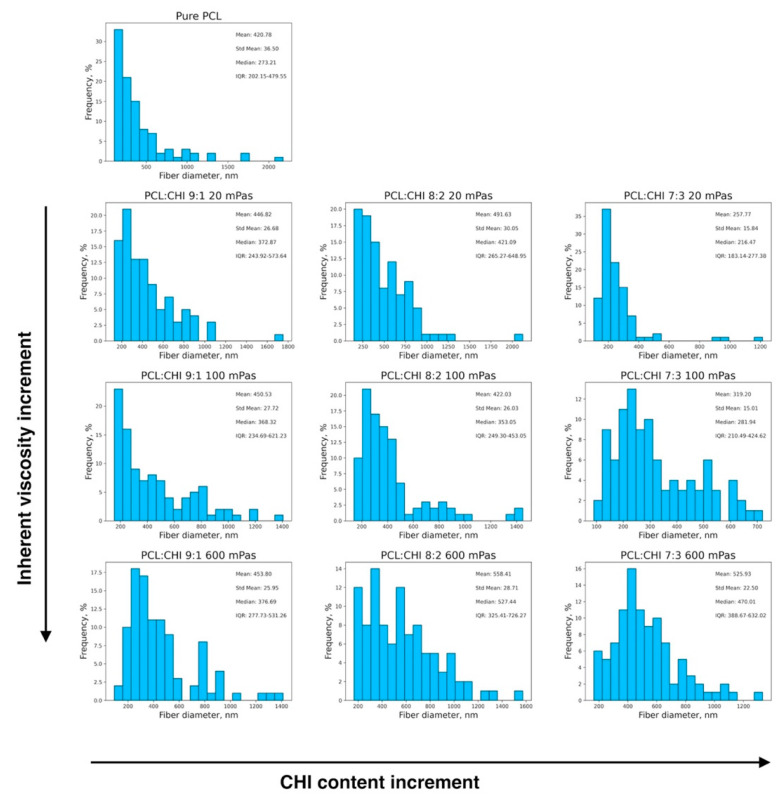
Histograms depicting the fiber diameter distributions of fabricated PCL and PCL/CHI biomaterials. Arrows denote directions in the increment of CHI content (from 10 to 30%) and CHI inherent viscosities (from 20 to 100 and 600 mPa·s) within the fibers.

**Figure 3:**
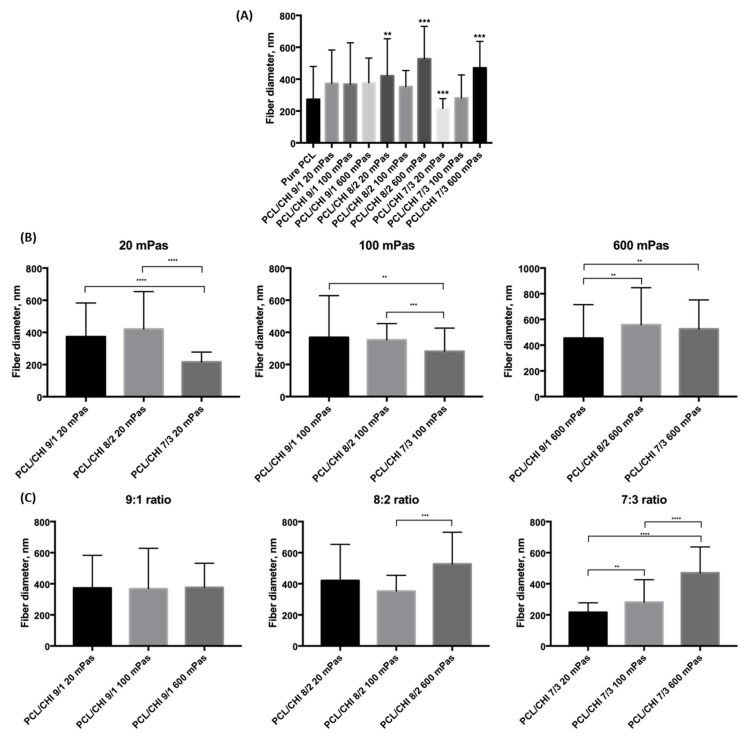
Results of multiple-comparison test of fabricated PCL and PCL/CHI biomaterials (**A**) in general, (**B**) for different CHI inherent viscosities (20, 100 and 600 mPa·s) and (**C**) for different PCL:CHI ratios (9:1, 8:2 and 7:3). (**A**) Asterisks denote statistically significant differences in the fiber diameter distributions compared to the distribution of pure PCL. Other asterisks denote statistically significant differences in the fiber diameter distribution within (**B**) viscosities or (**C**) PCL:CHI ratios with *p* < 0.05 significance level.

In all PCL and PCL/CHI samples, SEM images revealed the formation of homogeneous fibrous meshes with little to no beads or defects (Figure 1). Moreover, the majority of the diameters of the fibers of all the tested formulations were within the range of 250–450 nm. Nonetheless, fiber diameter analyses displayed significant variations among fabricated formulations by changing the PCL:CHI weight ratio (9:1 to 8:2 or 7:3) and inherent viscosities of CHI (20 to 100 or 600 mPa·s). In the following figures (Figure 2 and Figure 3), detailed statistical evaluations of the fiber diameter sizes and distributions are exhibited.

Additionally, fibrous mesh density was found to differ among the different tested parameters, which is related to the productivity of the ES process. Overall, it can be seen in Figure 1, Figure 2 and Figure 3 that the least dense fibrous meshes were obtained in samples fabricated with 600 mPa·s CHI inherent viscosity (PCL/CHI_9/1_600, PCL/CHI_8/2_600, PCL/CHI_7/3_600), whereas the densest fibrous layers were obtained in formulations synthesized with pure PCL and 20 mPa·s CHI inherent viscosity (PCL/CHI_9/1_20, PCL/CHI_8/2_20, PCL/CHI_7/3_20). The PCL/CHI_7/3_20 formulation showed the narrowest fiber diameter distribution (257.77 ± 15.84 nm), with more than 35% of the fibers in this range.

In Figure 3A, it can be observed that only four of the fabricated biomaterials were found to be statistically significantly different from the pure PCL formulation (*p* = 0.00051 for PCL/CHI_8/2_20, *p* = 7.964·10^−8^ for PCL/CHI_8/2_600, *p* = 0.00003 for PCL/CHI_7/3_20 and *p* = 2.967·10^−8^ for PCL/CHI_7/3_600). Moreover, the CHI content effect is studied in Figure 3B for each specific CHI inherent viscosity, again conducting multiple-comparison tests within each group of fabricated nanofibrous biomaterials. In this case, fiber diameter differences of between 9:1 and 7:3 ratios were found to be statistically significant for all tested CHI inherent viscosities.

Meanwhile, multiple-comparison tests were conducted within each PCL:CHI weight ratio of fabricated nanofibers in order to investigate the effect of CHI inherent viscosity (Figure 3C). Results for a 9:1 PCL:CHI weight ratio showed that nanofiber diameters were not statistically significant between 20, 100 and 300 mPa·s CHI inherent viscosities. However, a significant difference was found between 100 mPa·s and 600 mPa·s CHI for 8:2 PCL:CHI weight ratio. Additionally, statistically significant differences between each of the tested biomaterials were obtained for the 7:3 PCL:CHI weight ratio, resulting in the thickest fibers being in PCL/CHI_7/3_600. Overall, as the CHI amount increases, a more pronounced CHI viscosity effect is noticed.

**Figure 4:**
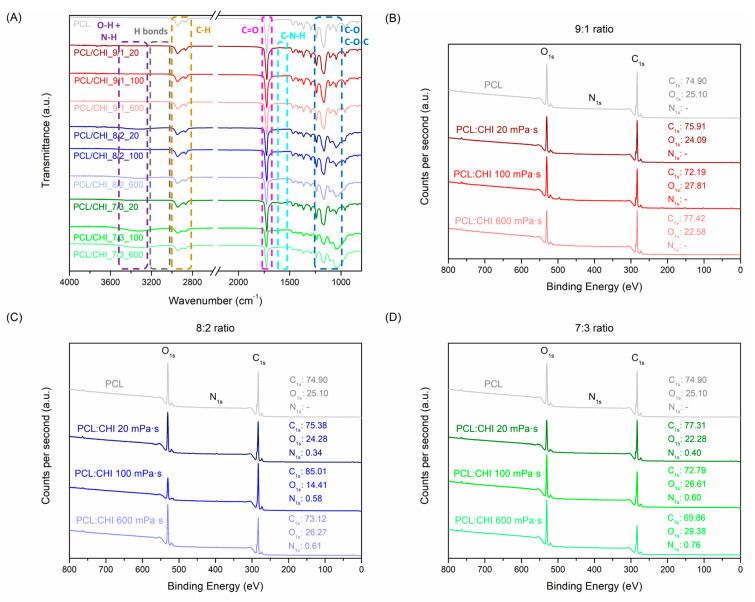
(**A**) FTIR spectra and XPS diagrams at (**B**) 9:1, (**C**) 8:2 and (**D**) 7:3 PCL:CHI weight ratios of PCL and PCL/CHI nanofibers.

Moreover, FTIR was employed to analyze the structure and interactions of PCL/CHI nanofibers (Figure 4A). The most typical vibration bands of PCL are C-H stretching (2850–3000 cm^−1^), C=O carbonyl stretching (1725 cm^−1^) and C-O-C plus C-O stretching (1050–1250 cm^−1^). Simultaneously, the CHI spectrum (Figure S1) possesses characteristic peaks at 3250–3500 cm^−1^ (O-H and N-H stretching), 2850–3000 cm^−1^ (C-H stretching), 1600–1750 cm^−1^ (amide I, C=O stretching), 1500–1580 cm^−1^ (amide II, NH bending), 1300–1400 cm^−1^ (amide III, C-N stretching) and 1050–1250 cm^−1^ (C-O-C and C-O stretching) [39,40].

After ACES, PCL and CHI combinations led to a significant increase in peak intensities at 1500–1580 cm^−1^ (amide II, N-H bending), 1300–1400 cm^−1^ (amide III, C-N stretching) and 1050–1250 cm^−1^ (C–O–C + C–O stretch). In addition, the appearance of a vibration band at 3250–3500 cm^−1^ (O-H and N-H stretching) also validated PCL/CHI nanofiber combinations owing to hydroxyl, amine and amide groups of CHI along with the appearance of intramolecular H-bonds within PCL and CHI chains. Such changes in PCL/CHI nanofiber vibration bands are more visible in combinations using high CHI quantities and viscosities, as observed in PCL/CHI_8/2_600, PCL/CHI_7/3_100 and PCL/CHI_7/3_600. Nevertheless, the C=O stretching (1600–1750 cm^−1^) vibration band did not suffer any notable intensity increment between PCL and PCL/CHI spectra due to almost imperceptible amide quantities in the CHI structure (deacetylation degrees > 98%). PCL, CHI and PCL/CHI absorption peak assignments were endorsed by prior FTIR analyses [41,42,43].

Similarly, the chemical composition of PCL and PCL/CHI nanofibers was quantitatively measured by XPS (Figure 4B,D, Table S1). As expected, PCL nanofibers only exhibited carbon and oxygen atomic compositions (74.90% C_1s_, 25.10% O_1s_). After combination of PCL with CHI at a 9:1 weight ratio (Figure 4B), atomic carbon and oxygen compositions did not vary significantly in PCL/CHI_9/1_20 (75.91% C_1s_, 24.09% O_1s_), PCL/CHI_9/1_100 (72.19% C_1s_, 27.81% O_1s_) and PCL/CHI_9/1_600 (77.42% C_1s_, 22.58% O_1s_) formulations.

Nonetheless, nitrogen atomic composition could be quantified in the remaining PCL/CHI nanofibers synthesized with a higher CHI quantity (Figure 4C,D, 8:2 and 7:3 PCL:CHI weight ratios), which are 0.34% N_1s_ for PCL/CHI_8/2_20, 0.58% N_1s_ for PCL/CHI_8/2_100, 0.61% N_1s_ for PCL/CHI_8/2_600, 0.40% N_1s_ for PCL/CHI_7/3_20, 0.60% N_1s_ for PCL/CHI_7/3_100 and 0.76% N_1s_ for PCL/CHI_7/3_600, respectively. So, a significant increase in nitrogen atomic composition (↑ % N) could be noticed as CHI quantity (7:3 > 8:2 > 9:1 PCL:CHI weight ratio) and inherent viscosity (600 > 100 > 20 mPa·s) increased, corroborating the successful fabrication of PCL/CHI nanofibers.

### 3.2. Nanomechanical Properties

**Figure 5:**
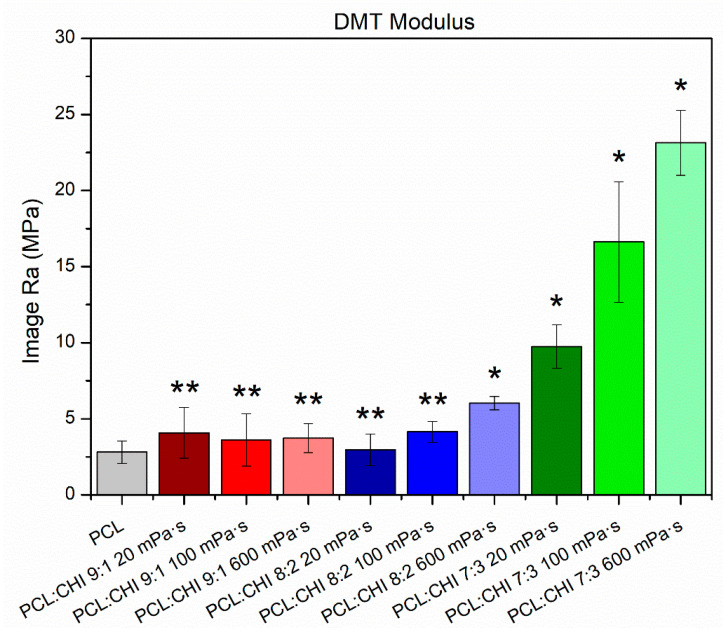
Nanomechanical properties (Young’s modulus, DMT model) of synthesized PCL and PCL/CHI nanofibers. ** Non-significant differences (*p* > 0.05); * significant differences (*p* < 0.05).

Mechanical properties of wound dressings are of key importance for maintaining a long-term high-quality treatment to patients. It has been reported that synthetic polymers (PCL) exhibited excellent mechanical properties, whereas natural polymers (CHI) possess more restrictive ones [44]. PCL/CHI electrospun dressings have displayed excellent mechanical properties in multiple previous scientific studies [45,46]. However, to the best of our knowledge, the nano-scale mechanical behavior of PCL/CHI nanofibers has never been reported previously. Consequently, the nanomechanical properties of nanofibers were determined by AFM (Figure 5 and Figure S2).

Nanofibers’ nanomechanical properties were highly dependent on the synthesis parameters used (Figure 5). In fact, the PCL sample possessed a Young’s modulus of 2.81 ± 0.74 MPa, while PCL/CHI formulations with low CHI quantities and viscosities exhibited a non-significant Young’s modulus increase (*p* > 0.05). Unexpectedly, PCL/CHI nanofibers fabricated with high CHI content and viscosities resulted in a significantly (*p* < 0.05) higher Young’s modulus, revealing an improvement in nanomechanical properties (6.02 ± 0.44 MPa for PCL/CHI_8/2_600, 9.75 ± 1.43 MPa for PCL/CHI_7/3_20, 16.63 ± 3.96 MPa for PCL/CHI_7/3_100 and 23.14 ± 2.15 MPa for PCL/CHI_7/3_600).

### 3.3. Contact Angle, Absorption Capacity and Biodegradation

An optimal wettability for polymeric dressings is required for effective wound healing [47]. In this regard, superhydrophobic (contact angle > 150°) wound dressings can be an ideal approach to enhance biological response, improve hemostasis and decrease bacterial adhesion [48]. However, pure PCL nanofibers are simply reported as hydrophobic biomaterials with 120–130° contact angle values [49]. Besides this, CHI has been historically used to increase wettability, which should make the obtention of superhydrophobic PCL/CHI nanofibers difficult [50].

**Figure 6:**
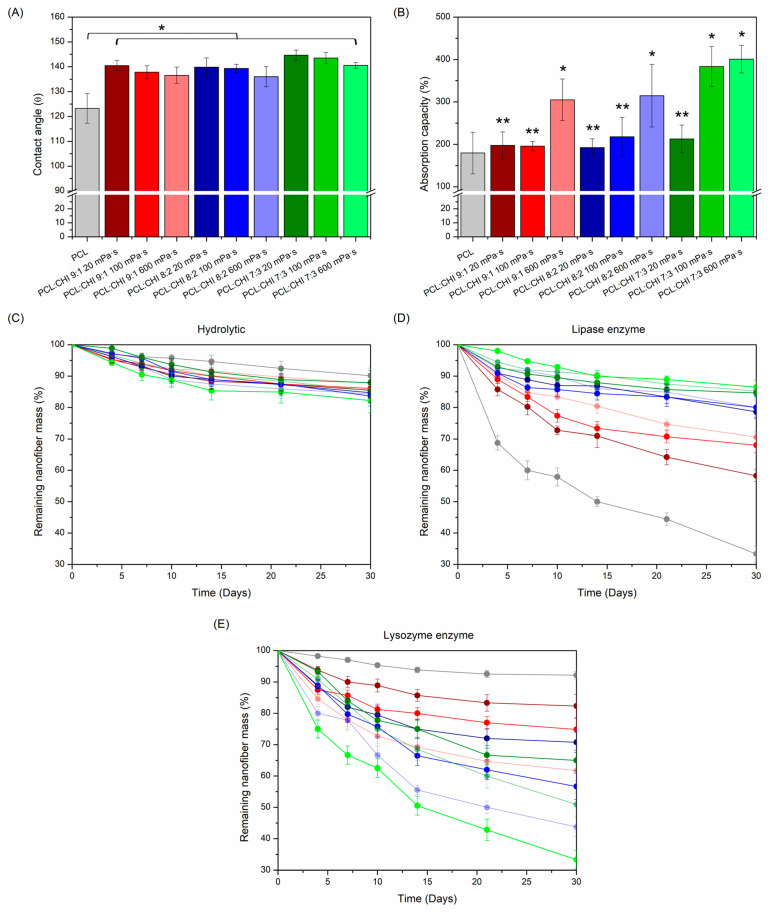
(**A**) Contact angle values; (**B**) absorption capacity (%); and (**C**) hydrolytic (PBS alone), (**D**) lipase enzymatic and (**E**) lysozyme enzymatic degradation profiles of electrospun PCL and PCL/CHI nanofibers. ** Non-significant differences (*p* > 0.05); * significant differences (*p* < 0.05).

Hence, the wettability of nanofibers was examined by contact angle measurements (Figure 6A). As expected, PCL nanofibers exhibited a contact angle of 123.25 ± 5.95° but, surprisingly, PCL/CHI nanofibers displayed a significant (*p* < 0.05) contact angle increase. In this way, PCL/CHI_7/3_100 and PCL/CHI_7/3_600 manifested the highest contact angle values (144.66 ± 2.06° and 143.52 ± 2.27°). Such superhydrophobicity could be attributed to asymmetric alignment with random orientation of nanofibers [51]. So, superhydrophobic PCL/CHI nanofibers could effectively prevent bacterial infections and induce hemostasis to promote wound healing.

In addition, absorption capacity is another functional property that primary dressings should fulfil to manage exudate excess. In fact, excessive exudate production can damage wounds and the surrounding skin (especially in chronic wounds), delaying healing by the prevention of cell migration [52,53]. In this regard, dressing absorbency can be evaluated in accordance with the UNE-EN ISO 13726-1:2002 standard [54]. Thereby, the exudate absorption capacity of fabricated PCL and PCL/CHI nanofibers was determined (Figure 6B).

As seen in Figure 6B, all PCL and PCL/CHI nanofiber formulations displayed a magnificent ability to absorb exudate. Actually, PCL nanofibers showed the lowest absorbency (179.58 ± 49.01%), whereas PCL/CHI nanofiber absorbency differed significantly (*p* < 0.05) as CHI quantity and viscosity increased. Hence, PCL/CHI_9/1_600, PCL/CHI_8/2_600, PCL/CHI_7/3_100 and PCL/CHI_7/3_600 samples can be categorized as superabsorbent dressings due to their outstanding exudate absorption capacities, which are 305.02 ± 48.93%, 314.58 ± 73.65%, 383.33 ± 47.14% and 400.89 ± 32.48%, respectively.

Although the combination of superhydrophobic PCL/CHI nanofibers and their subsequent great exudate absorption capacity may sound contradictory, it is a phenomenon that frequently occurs in the biomedical community and is described in the scientific literature [55,56]. Specifically, it is well known that superhydrophobic surfaces in continuous contact with an excess of water can provoke a significant decrease in contact angle values, resulting in the loss of nanofibers’ superhydrophobic nature. Fortunately, PCL/CHI nanofibers combine both beneficial properties for effective wound healing: initial superhydrophobicity, of which the durability is altered after a certain period of time to absorb detrimental excessive quantities of exudate.

Furthermore, in vitro degradation of synthesized PCL and PCL/CHI nanofibers was monitored in the absence (Figure 6C) and presence of lipase (Figure 6D) and lysozyme (Figure 6E) enzymes, which are especially used to biodegrade PCL and CHI. While lipase is known to hydrolyze ester bonds of PCL aliphatic polyester [57], lysozyme favors the hydrolysis of β-(1,4) glycosidic linkages of CHI [58]. As observed in Figure 6C, all PCL and PCL/CHI nanofiber formulations demonstrated great hydrolytic stability for 30 days (remaining nanofiber mass > 82%) without significant differences between all degradation profiles (*p* > 0.05).

However, PCL and PCL/CHI nanofiber biodegradability was magnified in lipase and lysozyme enzymatic mediums, with significant (*p* < 0.05) differences in the remaining nanofibers’ mass obtained practically from the third day until day 30. On the one hand, lipase medium (Figure 6D) led to the highest degradation rates of nanofibers with increased PCL content after 30 days: PCL (33.33 ± 2.55%), PCL/CHI_9/1_20 (58.27 ± 1.87%), PCL/CHI_9/1_100 (68.03 ± 2.33%) and PCL/CHI_9/1_600 (70.57 ± 1.70%). On the other hand, lysozyme (Figure 6E) caused the highest biodegradation in nanofibers with increased CHI quantity and viscosity, PCL/CHI_7/3_600 (32.38 ± 3.01%), PCL/CHI_8/2_600 (43.75 ± 2.96%), PCL/CHI_7/3_100 (50.89 ± 3.65%) and PCL/CHI_8/2_100 (56.67 ± 4.20%) being the least stable ones after 30 days.

Results so far reveal optimal physicochemical and nanomechanical properties along with ideal wettability, absorption capacity and stability of all PCL and PCL/CHI nanofibers for long-term applications as wound dressings. Therefore, the following biocompatibility, antibacterial and wound healing tests were preferentially performed only on PCL and PCL/CHI_7/3_600 formulations in order to validate their biological safety and bioactive properties.

### 3.4. Biocompatibility Test

Biological evaluation of a biomaterial intended for human use must follow the requirements of the ISO 10993-1:2010 standard, in which medical devices are classified under three main categories (surface, external communicating or implant device) and exposure duration (limited: t ≤ 24 h; prolonged: from 24 h to 30 days; and permanent: t > 30 days) [59]. Even so, regardless of the categorization assigned to the medical device, cytotoxicity and hemocompatibility assessments are common tests performed on dressings.

**Figure 7:**
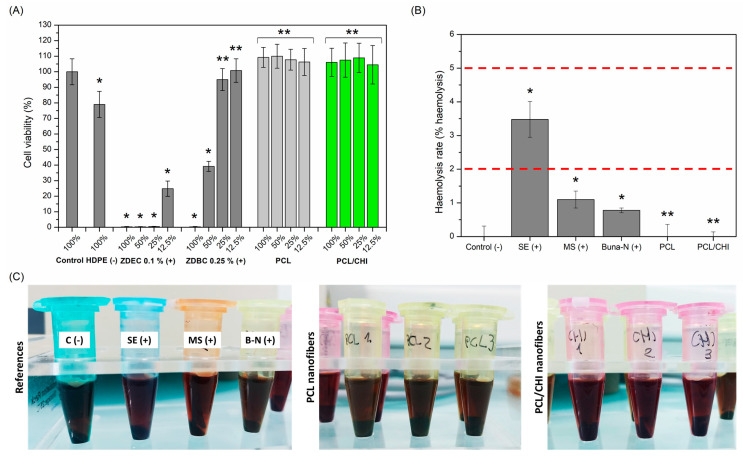
(**A**) Quantitative cell viability of L929 cells at 100%, 50%, 25% and 12.5% extract concentrations obtained by incubating nanofibers in culture media for 24 h at 37 °C. (**B**) Hemolysis rate (% hemolysis) values and (**C**) representative hemocompatibility photographs of controls and PCL and PCL/CHI nanofibers. ** Non-significant differences (*p* > 0.05); * significant differences (*p* < 0.05).

Therefore, biological safety of nanofiber-based dressings was first assessed by a cytotoxicity test (Figure 7A) according to the ISO 10993-5:2009 norm [36]. In this standard, it is stated that cell viability values below 70% are considered cytotoxic. In Figure 7A, it can be observed that cell viabilities of cytotoxic ZDEC positive controls (+) are inversely proportional to the extract concentration, whereas the non-cytotoxic HDPE negative control (−) displays a cell viability of 79.01 ± 8.42% (*p* < 0.05, blank cell viability: 100.00 ± 8.34%). What is more, both PCL and PCL/CHI nanofibers exhibited an adequate cytocompatibility with cell viabilities of 109.25 ± 6.43% for PCL and 106.07 ± 9.05% for PCL/CHI_7/3_100 at 100% concentration.

Conversely, hemocompatibility of nanofiber dressings was evaluated by quantifying hemolysis rate (Figure 7B,C), which is defined as the process in which hemoglobin is released from erythrocytes, either by destruction or through a partially damaged cell membrane. According to ISO 10993-4, blood-contacting biomaterials can be classified as hemolytic (hemolysis > 5%), slightly hemolytic (hemolysis between 2 and 5%) and nonhemolytic (hemolysis < 2%) [37]. So, both PCL and PCL/CHI samples could be categorized as nonhemolytic (0.00 ± 0.36% and 0.00 ± 0.14% hemolysis rates), demonstrating remarkable hemocompatibility. Indeed, electrospun nanofibers improve hemolysis rates of positive controls (0.78 ± 0.07 for Buna-N, 1.10 ± 0.25 for MS, 3.48 ± 0.53 for SE), even reaching the hemolytic activity of unexposed blood negative control (0.00 ± 0.31%): that is, an absence of hemolysis.

### 3.5. In Vitro Antibacterial Activity

**Figure 8:**
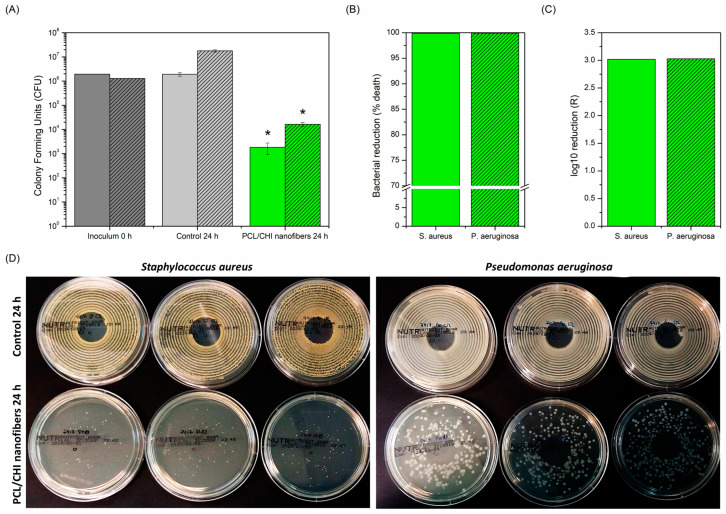
(**A**) CFU, (**B**) bacterial reduction (% death) and (**C**) log10 reduction (R) antibacterial activity of PCL/CHI nanofibers against *S. aureus* (no pattern) and *P. aeruginosa* (striped pattern) after 24 h. (**D**) Photographs of *S. aureus* and *P. aeruginosa* bacterial colony agar plates are also shown to corroborate quantitative results. * Significant differences (*p* < 0.05).

Antibacterial activity against infections is an important feature that nanofiber-based biomaterials should fulfil for successful wound healing [60]. It is well known that bacterial infections could promote exudate formation and delay the wound healing process, *S. aureus* and *P. aeruginosa* bacteria strains being the most common microorganisms isolated from chronic wound infections [61]. Such infections can be avoided by reducing the number of pathogens on wound sites through the employment of electrospun CHI-based nanofibers with antibacterial properties [62]. For this reason, antibacterial activity of PCL/CHI_7/3_100 was evaluated against *S. aureus* and *P. aeruginosa* after 24 h (Figure 8) since it is considered most critical for bacteria adhesion and proliferation [63].

Figure 8 displays the remarkable antibacterial activity of PCL/CHI_7/3_100 nanofibers against both *S. aureus* and *P. aeruginosa* due to a significant reduction in the number of viable bacteria compared to the control group (1.84·10^3^ ± 0.91·10^3^ CFU vs. 1.91·10^6^ ± 3.15·10^5^ CFU for *S. aureus* and 1.64·10^4^ ± 3.19·10^3^ CFU vs. 1.78·10^7^ ± 1.93·10^6^ CFU for *P. aeruginosa*). Therefore, PCL/CHI_7/3_100 showed nearly 100% bacteria reduction (99.90% for *S. aureus* and 99.91% for *P. aeruginosa*) with resultant log10 reductions (R) of 3.02 and 3.03, respectively. This way, the antibacterial activity of fabricated PCL/CHI nanofibers against *S. aureus* and *P. aeruginosa* is demonstrated for its potential application in chronically infected wound sites.

### 3.6. In Vitro Wound Healing Cell Proliferation Assay

Wound healing is a complex biological process in which tissue integrity is restored. Physiologically, tissue repair is composed of four main phases: hemostasis, inflammation, proliferation and remodeling [64]. While the first two stages involved in tissue repair last a maximum of 48 h, the proliferative stage begins in the microenvironment of the lesion within the first 48 h and can unfold up to 14 days after the onset of the lesion [65].

**Figure 9:**
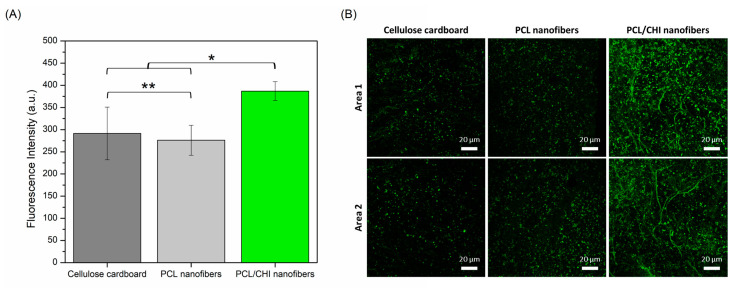
(**A**) Quantitative total fluorescence intensity (a. u.) calculated by ImageJ software obtained from (**B**) fluorescence images obtained by CLSM of cellulose cardboard, PCL nanofibers and PCL/CHI nanofibers after 48 h (2.5× magnification, 20 µm scale bar). Areas 1 and 2 are two representative randomly taken microphotographs of its well. ** Non-significant differences (*p* > 0.05); * significant differences (*p* < 0.05).

For this reason, proliferation not only is undoubtedly the most important stage of the healing process, but also is the most responsible for lesion closure itself including angiogenesis (restoration of vascular network), fibroplasia (formation of granular/fibrous tissue), re-epithelialization (covering of wound surface by the reconstruction of injured epithelium) and retraction (decrease in tissue area needing to heal) [66]. Therefore, the wound healing activity of fabricated nanofibers was evaluated by a cell proliferation assay using CLSM measuring the fluorescence intensity of green-stained HDFs (Figure 9). Cellulose cardboard in which nanofibers are deposited during the ACES process was used as a control.

As seen in Figure 9, cellulose cardboard and PCL nanofibers stimulated discrete proliferation ability in which HDFs proliferated equally comfortably (*p* > 0.05) in both samples (291.38 ± 59.52 and 276.11 ± 33.81 fluorescence intensities). Such proliferation results are in accordance with the scientific literature in which, although PCL nanofiber cell proliferative ability is considered adequate enough, the proliferation process can be promoted after combination with other bioactive polymers like CHI. Indeed, significantly (*p* < 0.05) more HDFs were found in PCL/CHI nanofibers (386.90 ± 21.17 fluorescence intensity) compared to PCL-only nanofibers, proving their enhanced wound healing activity.

## 4. Conclusions

Physicochemical and nanomechanical results confirmed the hypothesized possibility of combining PCL and CHI to synthesize nanofiber-based wound dressings. Furthermore, cytocompatibility and hemocompatibility results proved the biological safety of nanofibers. Moreover, PCL/CHI nanofibers exhibited antibacterial activity against *S. aureus* and *P. aeruginosa*, which undoubtedly would be beneficial to regenerate damaged tissues. Finally, the enhanced wound healing activity of PCL/CHI nanofibers was confirmed thanks to their ability to promote cell proliferation. All in all, bioactive PCL/CHI nanofiber-based dressings could be considered as potential candidates for long-term applications such as wound healing medical devices.

## Figures and Tables

**Table 1:** Sample codification of the fabricated PCL and PCL/CHI nanofibers with ACES.

Sample	PCL wt%	CHI wt%	Solvent System (Weight Ratio)	PCL:CHI Weight Ratio	CHI Viscosity in 1% Acetic Acid (mPa·s)
PCL (●)	10	2	FA:AA:Ac (1:1:1)	-	-
PCL/CHI_9/1_20 (●)				9:1 (red)	20 (dark)
PCL/CHI_9/1_100 (●)					100 (regular)
PCL/CHI_9/1_600 (●)					600 (light)
PCL/CHI_8/2_20 (●)				8:2 (blue)	20 (dark)
PCL/CHI_8/2_100 (●)					100 (regular)
PCL/CHI_8/2_600 (●)					600 (light)
PCL/CHI_7/3_20 (●)				7:3 (green)	20 (dark)
PCL/CHI_7/3_100 (●)					100 (regular)
PCL/CHI_7/3_600 (●)					600 (light)

## Data Availability

Data are contained within the article.

## Supplementary Materials

The following supporting information can be downloaded at: https://www.mdpi.com/article/10.3390/polym16101333/s1, Figure S1: FTIR spectra of pure CHI used to fabricate PCL/CHI nanofibers; Table S1: Atomic compositions (%) of electrospun PCL and PCL/CHI nanofibers obtained by XPS; Figure S2: High-resolution AFM channels with obtained nanomechanical properties of synthesized PCL and PCL/CHI nanofibers.
