# Supplementary material for: Alternating Current Electrospinning of Polycaprolactone/Chitosan Nanofibers for Wound Healing Applications
Source: Polymers (Basel). 2024 May 9;16(10):1333. doi: 10.3390/polym16101333 (PMC11125242; doi:10.3390/polym16101333)
Supplement: Supplementary file 1 [file polymers-16-01333-s001.zip › polymers-2960712-supplementary.pdf]

# Supplementary material

# Alternating Current Electrospinning of Polycaprolactone/Chitosan Nanofibers for Wound Healing Applications

**Jon Andrade del Olmo [1,*], Petr Mikeš [2], Nikifor Asatiani [2], José María Alonso [1], Virginia Sáez Martínez [1] and Raúl Pérez González [1]**

[1] i+Med S. Coop., Alava Technology Park, Albert Einstein 15, nave 15, 01510 Vitoria-Gasteiz, Spain; jalonso@imasmed.com (J.M.A.); vsaez@imasmed.com (V.S.M.); rperez@imasmed.com (R.P.G.)

[2] Department of Physics, Faculty of Science, Humanities and Education, Technical University of Liberec, Studentská 1402/2, 461 17 Liberec, Czech Republic

* Correspondence: jandrade@imasmed.com

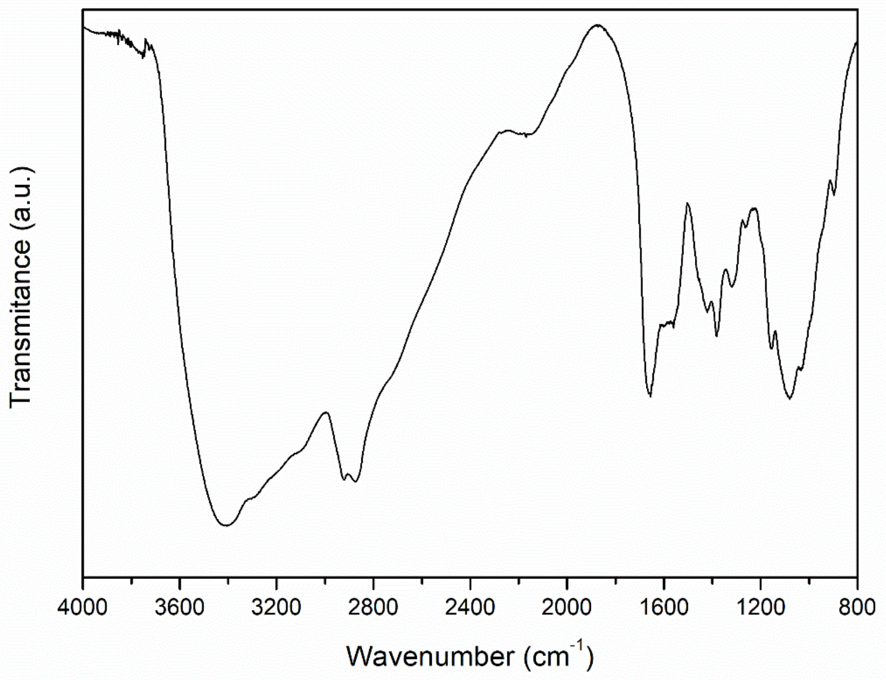


**Figure S1.** FTIR spectra of pure CHI used to fabricate PCL/CHI nanofibers.

**Table S1**. Atomic compositions (%) of electrospun PCL and PCL/CHI nanofibers obtained by XPS.

| Sample | Atomic composition (%) | | |
|---|---|---|---|
| | $C_{1s}$ | $O_{1s}$ | $N_{1s}$ |
| PCL (●) | 74.90 | 25.10 | - |
| PCL/CHI_9/1_20 (●) | 75.91 | 24.09 | - |
| PCL/CHI_9/1_100 (●) | 72.19 | 27.81 | - |
| PCL/CHI_9/1_600 (●) | 77.42 | 22.58 | - |
| PCL/CHI_8/2_20 (●) | 75.38 | 24.28 | 0.34 |
| PCL/CHI_8/2_100 (●) | 85.01 | 14.41 | 0.58 |
| PCL/CHI_8/2_600 (●) | 73.12 | 26.27 | 0.61 |
| PCL/CHI_7/3_20 (●) | 77.31 | 22.28 | 0.40 |
| PCL/CHI_7/3_100 (●) | 72.79 | 26.61 | 0.60 |
| PCL/CHI_7/3_600 (●) | 69.86 | 29.38 | 0.76 |

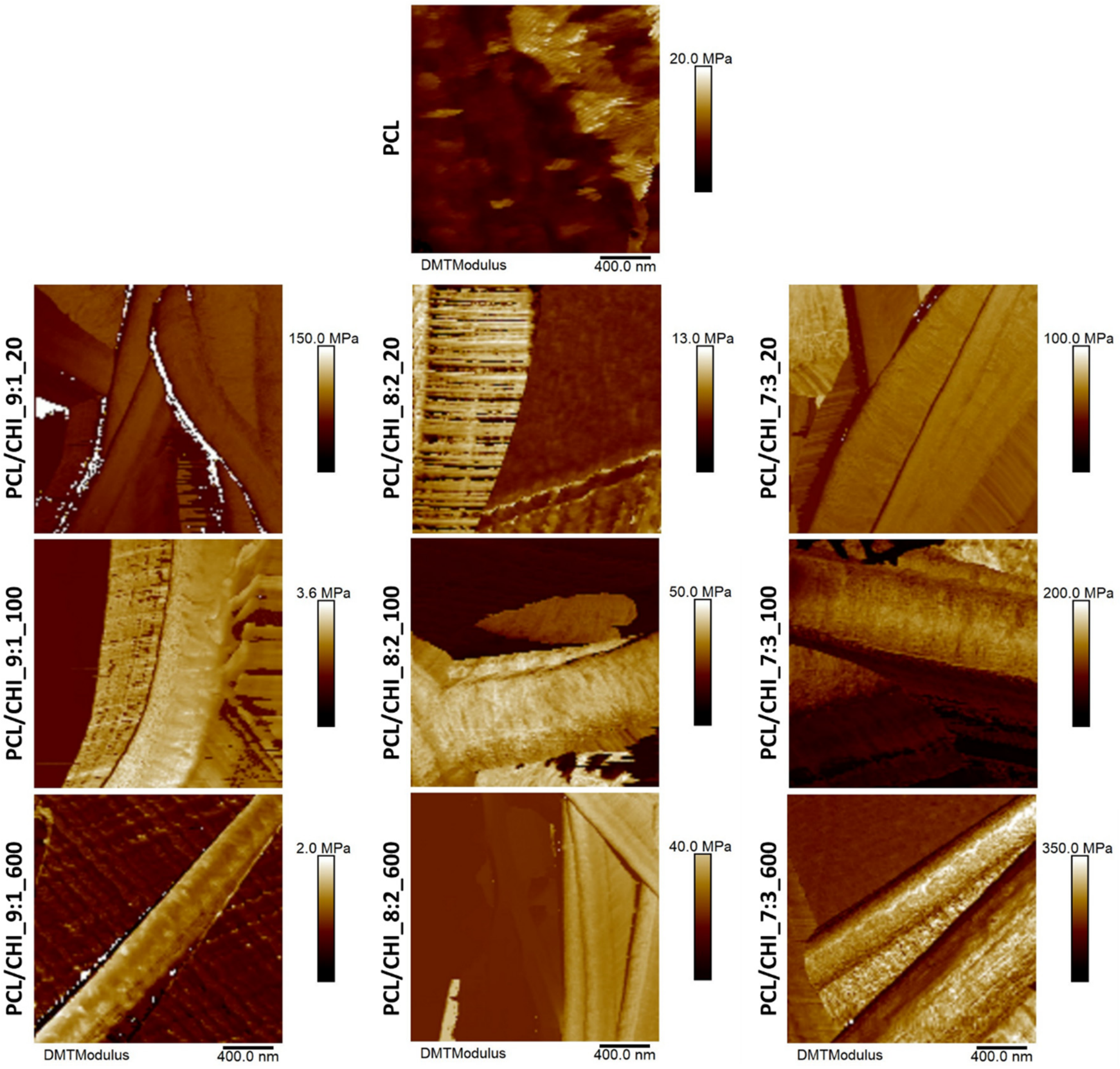


**Figure S2.** High-resolution AFM channels with obtained nanomechanical properties of synthesized PCL and PCL/CHI nanofibers.
